# Reduced Elastin Fibers and Melanocyte Loss in Vitiliginous Skin Are Restored after Repigmentation by Phototherapy and/or Autologous Minigraft Transplantation

**DOI:** 10.3390/ijms232315361

**Published:** 2022-12-06

**Authors:** Tomohisa Hirobe, Hisao Enami

**Affiliations:** Department of Histology and Pathology, Shinjuku Skin Clinic, 3-24-1 Shinjuku, Shinjuku-ku, Tokyo 160-0022, Japan

**Keywords:** melanocyte, melanoblast, differentiation, epidermis, dermis, elastin, vitiligo, repigmentation, ultraviolet light, skin transplantation

## Abstract

Vitiligo is a hypopigmentation disease characterized by melanocyte death in the human epidermis. However, the mechanism of vitiligo development and repigmentation is largely unknown. Dermal fiber components might play an important role in vitiligo development and repigmentation. Indeed, our preliminary study demonstrated that elastin fibers were decreased in vitiliginous skin, suggesting that the elastin fiber is one of the factors involved in vitiligo development and repigmentation. To confirm our hypothesis, we investigated whether elastin fibers can be restored after treatment using phototherapy and/or autologous skin transplantation. Punch biopsies from 14 patients of stable nonsegmental vitiligo vulgaris were collected from nonlesional, lesional, and repigmented skin, and processed to dopa and combined dopa–premelanin reactions. Melanocytes positive to the dopa reaction and melanoblasts/melanocytes positive to the combined dopa–premelanin reaction were surveyed. Moreover, elastin fibers were detected by Victoria blue staining. Numerous melanocytes and melanoblasts were observed in the epidermis of repigmented skin after the treatment. Moreover, in the dermis of repigmented skin, elastin fibers were completely recovered or even upregulated. These results suggest that melanocyte loss in the vitiliginous skin, as well as melanocyte differentiation in repigmented skin, may be at least in part regulated by elastin fibers in the dermis.

## 1. Introduction

Melanocytes differentiate from neural crest-derived melanoblasts [1] and produce melanin-laden melanosomes [2,3] and then transport them to surrounding keratinocytes. Melanocytes contribute to the human skin color by regulating the content and ratio of eumelanin and pheomelanin present in melanosomes [3]. Vitiligo is a hypopigmentation disease characterized by achromatic or hypochromatic macules in various sites of human skin. Affected lesions are characterized by the absence of melanin and functioning melanocytes [4,5]. Although the mechanism of vitiligo development has not been fully understood, melanocyte death by CD8^+^ T cells may be one of the most prevailing hypotheses [4,5]. Another hypothesis is that the decrease in growth factors and cytokines derived from keratinocytes [1,6,7] and fibroblasts [1,8] fails to support melanocyte proliferation and differentiation, resulting in melanocyte loss in the skin. However, there has been little attention on the role of dermal fibers, such as collagen and elastin fibers, in the development and repigmentation of vitiligo.

Collagen is one of the major dermal components and is involved in regulating the mechanical stability of the skin [9]. Elastin fibers are involved in regulating the elasticity and strength of skin in cooperation with collagen fibers [10,11]. A unique hexapeptide, Val-Gly-Val-Ala-Pro-Gly (VGVAPG), is repeated multiple times in human elastin molecules [10]. 

Our preliminary study demonstrated that elastin fibers were decreased in vitiliginous skin, suggesting the possibility that elastin fibers are involved in vitiligo development and repigmentation [12]. In this study, to confirm our hypothesis, we investigated whether the reduced elastin fibers and melanocyte loss in vitiliginous skin can be restored after phototherapy and/or skin transplantation. Phototherapies using psoralen–ultraviolet light A (PUVA) [13], monochromatic excimer light (MEL, 308 nm) [14], and narrow band-UVB (NB-UVB, 311 nm) [15], skin transplantation, such as autologous machine-using minigrafts (mMG) [16,17], and transplantation of cultured/non-cultured epidermal cell suspensions [18], have been reported to induce repigmentation in vitiliginous skin. However, it is unknown whether the phototherapy and/or mMG can restore the reduced elastin fibers and melanocyte loss in the vitiliginous skin. Thus, we investigated in detail whether the reduced elastin fibers and melanocyte loss in vitiliginous skin can be restored after the treatment with phototherapy (MEL/NB-UVB) and/or mMG. Our results suggest that the reduced elastin fibers and melanocyte loss in vitiliginous skin can be restored after phototherapy and/or mMG.

## 2. Results

### 2.1. Repigmentation of Lesional Skin after Phototherapy and/or mMG

**Figure 1:**
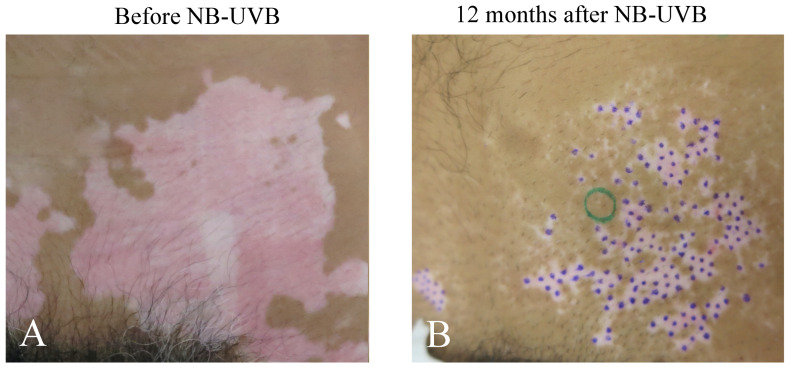
Appearance of lesional skin (lower abdomen, patient No. 1) before (**A**) and after (**B**) exposures with NB-UVB. After 12 months, excellent repigmentation was observed. Repigmented areas are present in the patch within the lesional skin (perifollicular repigmentation pattern). Skin biopsy of repigmented area (green circle) was taken (**B**). Small violet dots indicate that the white macules persisted even after the long NB-UVB phototherapy. Green circle shows regimented skin area and 3 skin cites were taken from here for histochemical analysis.

Excellent repigmentation was observed in 14 cases (Table 1) 0.7 to 24 months after exposures with 1 to 2 minimal erythema doses (MEDs) with ascending exposure with a 10–15% increase in the dose after checking repigmentation of MEL and/or NB-UVB with or without mMG. All were “perifollicular” repigmentation patterns (pigmented spots occurred around the hair pouch at several places in the lesional skin, Figure 1). Average number of sessions (exposures) of single treatment (MEL or NB-UVB) was 65.2 ± 21.5 session (*n* = 5) and that of double treatment (MEL + NB-UVB) was 24.8 ± 6.1 sessions (*n* = 9), respectively. This difference is statistically significant (*p* < 0.05). Average month of exposure of single treatment is 11.8 ± 3.3 months (*n* = 5), while that of double treatment is 3.9 ± 1.0 months (*n* = 9). This difference is also statistically significant (*p* < 0.05). Average dose of exposure of single treatment is 41.3 ± 9.9 J/cm^2^ (*n* = 5), while that of double treatment is 13.2 ± 4.9 J/cm^2^ (*n* = 9). This difference is also statistically significant (*p* < 0.05). These results suggest that the single treatment modality requires more sessions, times, and doses to obtain repigmentation.

### 2.2. Melanocytes and Melanoblasts in Nonlesional, Lesional, and Repigmented Skin

**Figure 2:**
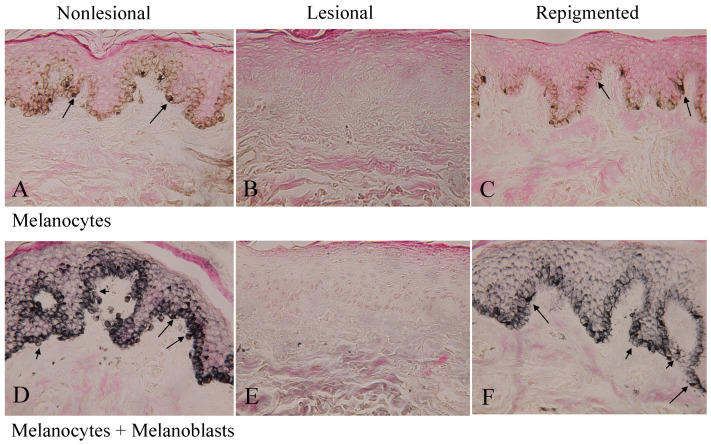
The dopa reaction of nonlesional belly skin (**A**) of patient No. 1 reveals many tyrosinase-positive melanocytes (long arrows) with well-developed dendrites. Melanoblasts (short arrows) and melanocytes (long arrows) positive to the combined dopa–premelanin reaction are also observed (**D**). By contrast, no melanocytes (**B**) and melanoblasts (**E**) are observed in the lesional skin. However, many dendritic melanocytes (**C**, long arrows) and melanoblasts (short arrows, **F**)/melanocytes (long arrows, **F**) are observed in the repigmented skin. Although epidermal melanin pigmentation in the repigmented skin is lower than in the nonlesional skin, the number of melanocytes is comparable to that of nonlesional skin. Original magnification level, ×400.

The dopa reaction (Figure 2A) of nonlesional skin of patient No. 1 (Figure 1A) revealed many melanocytes with well-developed dendrites, and the combined dopa–premelanin reaction (Figure 2D) revealed melanocytes and melanoblasts. Melanoblasts are lightly stained by the combined dopa–premelanin reaction, while melanocytes are darkly stained by the reaction. By contrast, no melanocytes (Figure 2B) and melanoblasts (Figure 2E) were observed in the lesional skin. However, many dendritic melanocytes (Figure 2C) and melanoblasts/melanocytes (Figure 2F) were observed in the repigmented skin 12 months after single phototherapy (NB-UVB). Although epidermal melanin pigmentation in the repigmented skin was lower than in the nonlesional skin, the number of melanocytes was comparable to that of nonlesional skin, suggesting that the skin color may be the result of epidermal pigmentation rather than the presence of substantial number of epidermal melanocytes. In the skin of the other 13 patients, similar results were obtained.

### 2.3. Collagen and Elastin Fibers in Nonlesional, Lesional, and Repigmented Skin

**Figure 3:**
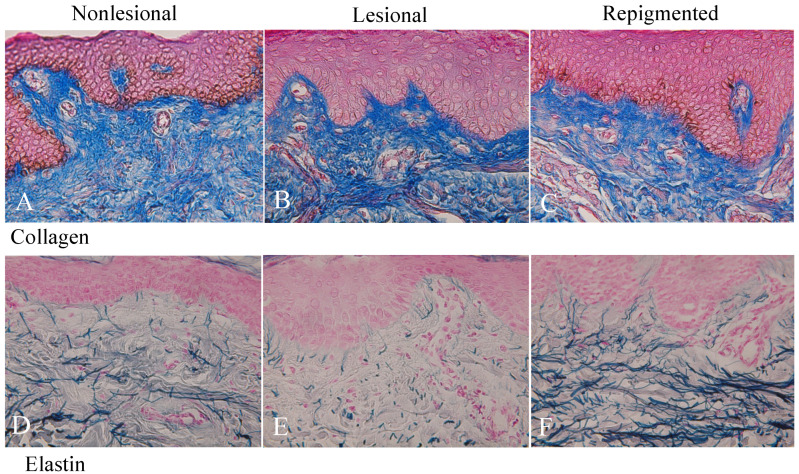
Azan staining reveals well-developed collagen fibers (stained light blue) in the nonlesional (**A**), lesional (**B**), and repigmented (**C**) skin in a similar density. The elastin staining reveals well-developed elastin fibers (stained blue-black) in the nonlesional skin (**D**), though in the lesional skin, elastin fibers (**E**) are greatly decreased. However, in the repigmented skin (**F**), elastin fibers are dramatically increased and exceeded compared with the level of nonlesional skin. Original magnification level, ×400.

Collagen fibers were equally distributed in nonlesional (Figure 3A), lesional (Figure 3B), and repigmented (Figure 3C) skin of patient No 1. Collagen fibers were not decreased after the treatment except in the two females (No. 3 and No. 12, Table 1). However, elastin fibers in the lesional skin (Figure 3E) were greatly reduced and the fibers were torn and sparse compared with nonlesional skin (Figure 3D) in all cases (Table 1). However, elastin fibers in the repigmented skin (Figure 3F) were dramatically increased and exceeded those in the nonlesional skin. The elastin fibers became thick and reached the bottom of the rete ridge and inter-rete ridge epidermis (Figure 3F). Moreover, the tip of the elastin fibers extended the basal layer of the epidermis. In the skin of the other 13 patients, similar results were obtained.

### 2.4. Differences between Nonlesional and Repigmented Skin in the Number of Epidermal Melanocytes and Melanoblasts

Although the number of melanocytes in the nonlesional and repigmented skin differed between skin sites, the averages of the two populations were 111.6 ± 6.1 cells/0.1 mm^2^ and 133.9 ± 14.9 cells/0.1 mm^2^, respectively (Table 1). This difference was not statistically significant. On the other hand, the number of melanoblasts in the nonlesional skin did not fluctuate and the average was 103.2 ± 1.8 cells/0.1 mm^2^. The average number of melanoblasts in the repigmented skin was 117.1 ± 13.4 cells/0.1 mm^2^. This difference was also not statistically significant. These results suggest that the averages of epidermal melanocytes and melanoblasts in the repigmented skin are completely restored.

### 2.5. Differences between Single and Double Phototherapies in the Recovery of Epidermal Melanocytes/Melanocytes and Elastin Fibers in the Repigmented Skin

Although the average numbers of epidermal melanocytes and melanoblasts in the repigmented skin after double phototherapy tended to be greater than those of single phototherapy, these differences were not statistically significant (Table 2A). The percentages of full recovery (Table 1, ↑↑↑) and moderate recovery (Table 1, ↑↑) of elastin fibers after single phototherapy were 80.0% (4/5) and 20.0% (1/5), respectively, and those of double phototherapy were 66.7% (6/9) and 33.3% (3/9), respectively. These differences were not statistically significant. These results suggest that the recovery of melanocytes/melanocytes and elastin fibers in the repigmented skin does not differ between single and double phototherapies.

### 2.6. Differences between Phototherapy Alone and Phototherapy plus mMG in the Recovery of Epidermal Melanocytes/Melanocytes and Elastin Fibers in the Repigmented Skin

Although epidermal melanocytes and melanoblasts in the repigmented skin after phototherapy plus mMG tended to be greater than those after phototherapy alone, these differences were not statistically significant (Table 2B). The percentages of full recovery and moderate recovery of elastin fibers after phototherapy alone were 66.7% (4/6) and 33.3% (2/6), respectively, and those of phototherapy plus mMG were 75.0% (6/8) and 25.0% (2/8), respectively (Table 1). However, these differences were not statistically significant. These results suggest that the recovery rate of epidermal melanocytes/melanocytes and elastin fibers in the repigmented skin does not differ between phototherapy alone and phototherapy plus mMG.

### 2.7. Gender Differences in the Recovery of Epidermal Melanocytes/Melanocytes and Elastin Fibers in the Repigmented Skin after Phototherapy and/or Phototherapy plus mMG

Although the average numbers of epidermal melanocytes and melanoblasts in the repigmented skin of males tended to be greater than those of females, these differences were not statistically significant (Table 2C). The percentages of full recovery and moderate recovery of elastin fibers in the repigmented skin of females were 71.4% (5/7) and 28.6% (2/7), respectively, while those in the males were 71.4% (5/7) and 28.6% (2/7), respectively (Table 1). These results suggest that the recovery of epidermal melanocytes/melanocytes and elastin fibers in the repigmented skin after phototherapy and phototherapy plus mMG do not differ between females and males.

### 2.8. Age Differences in the Recovery of Epidermal Melanocytes/Melanocytes and Elastin Fibers in the Repigmented Skin after Phototherapy and Phototherapy plus mMG

Although the average number of epidermal melanocytes in the repigmented skin of the young tended to be greater than those of the old, this difference was not statistically significant (Table 2D). However, the difference between the young and old in the number of epidermal melanoblasts was statistically significant (Table 2D, *p* < 0.05). On the other hand, the percentage of full recovery and moderate recovery of elastin fibers in the repigmented skin of the young (6–15 years) was 42.9% (3/7) and 57.1% (4/7), respectively, while that of the old (31–77 years) was 100% (7/7) and 0% (0/7), respectively (Table 1). This difference was statistically significant (Table 1, *p* < 0.05). These results suggest that in the repigmented skin in the young, epidermal melanoblasts are more greatly increased than in the old, but the recovery of elastin fibers is more delayed in the young than in the old. 

## 3. Discussion

The present study demonstrated that in repigmented skin after phototherapy and/or mMG to the vitiliginous skin, the loss of melanocytes and melanoblasts in the epidermis was fully restored and the reduced elastin fibers in the dermis was also restored or more upregulated irrespective of gender difference. Moreover, the density, length, and thickness of elastin fibers were increased by these treatments. By contrast, collagen fibers were not decreased after the treatment except in two cases. These results suggest that elastin fibers but not collagen fibers are involved in regulating melanocyte death and redifferentiation in the vitiliginous skin.

It is unknown which cells present in vitiliginous skin or the skin of minigraft are involved in the redifferentiation of melanocytes. Are these cells melanocyte stem cells present in the bulge area of hair follicles (hair bulge) or melanocyte precursors present in the interfollicular epidermis? It is established that, in mice, melanocyte stem cells are located in the hair bulge [19]. They reproduce transit amplifying cells, including melanoblasts. In human skin, however, it is not yet established that melanocyte stem cells are located in the hair bulge, since transgenic technology is not applicable to humans. Moreover, skin structure in humans greatly differs from that in mice, as human skin is largely glabrous and the interfollicular epidermis possesses large rete ridges where numerous melanoblasts and melanocytes are present even in adults [20]. On the other hand, murine epidermal melanocytes are present only one month after birth, except in the tail, foot pad, and ears [1]. Recently, a study using molecular markers of melanocyte stem cells or precursor melanocytes has been reported in humans. Goldstein et al. [21] reported that human melanocyte stem cells are located in the infundibula and bulge area of hair follicles. These cells were detected as cells positive to tyrosinase-related protein 2 (TYRP2 or dopachrome tautomerase, DCT) but not tyrosinase and KIT. They also reported that melanocyte precursors positive to KIT but not tyrosinase and TYRP2 were present in the vitiliginous epidermis after exposure with NB-UVB but not in normal epidermis. However, Grichnik et al. [22] reported the presence of undifferentiated cells in the epidermis positive to KIT and B-cell lymphoma 2 (BCL-2) but not TYRP1. Recently, Michalak-Micka [23] reported that KIT^+^CD90^–^ and KIT^+^CD90^+^ cells were dendritic melanocytes, whereas KIT^–^CD90^+^ cells were bipolar cells with limited amounts of melanin in the human interfollicular epidermis. These KIT^–^CD90^+^ cells lacked the expressions of human melanoma black 45 (HMB45), tyrosinase, and TYRP1. They proposed that these KIT^–^CD90^+^ cells are precursor reservoir melanocytes in the human epidermis. Our previous study [20] also revealed that melanocytes and melanoblasts (no tyrosinase activity and melanin depositions) are present in the epidermis in the rete ridge and inter-rete ridge using combined dopa–premelanin staining [1]. Taken together, these results suggest that precursor melanocytes are present in the interfollicular epidermis of human skin and constitute reservoir cells for the differentiation of melanocytes, even though the presence of melanocyte stem cells are not established in the human interfollicular epidermis. It is assumed that melanocyte stem cells present in the hair bulge or melanocyte precursors (KIT^+^BCL-2^+^TYRP1^–^cells or KIT^–^CD90^+^ cells) present in the interfollicular epidermis are involved in regulating the redifferentiation of melanocytes after phototherapy and/or mMG. However, this assumption remains to be confirmed.

The melanocyte and melanoblast populations and the density of elastin fibers seem to reach normal levels after phototherapy and/or mMG. During this repigmentation process, the proliferation and differentiation of melanoblasts and melanocytes in the lesional skin or grafted skin are thought to be induced or increased, being accompanied with elastin fiber reconstruction. Thus, it can be assumed that the proliferation and differentiation of melanocytes and melanoblasts well correlate with the upregulation and downregulation of elastin fiber constructions. Our previous study [24] also demonstrated that the treatment of elastin peptides and ferrous ferric chloride [1] to normal human skin stimulated the proliferation and differentiation of epidermal melanocytes. In animals, Chang et al. [25] reported that elastin peptides stimulated the proliferation and differentiation of mouse melanoblasts/melanocytes. Taken together, these results may suggest that elastin is one of the important molecules involved in the proliferation and differentiation of epidermal melanocytes in normal skin of mice, as well as the normal and vitiliginous skin of humans.

Ono et al. [26] reported that, in dermal melanocytosis, dendrites of human melanocytes were aligned along the long axis of elastin fibers. Moreover, the same intimacy was reported between elastin fibers and normal dermal melanocytes of monkeys (*Cynomolgus macaques*). Chang et al. [25] reported that normal embryonic mouse melanoblasts expressed elastin binding protein, and VGVAPG peptides stimulated the dendritogenesis of mouse differentiated melanocytes derived from neural crest cell cultures. Moreover, they reported that the interaction between melanoblasts and elastin binding protein was initiated early in the skin development (E12.5). In the present study, elastin fibers were dramatically decreased in the vitiliginous skin, whereas those in the repigmented skin were greatly increased, became thick, and extended to the basal layer epidermis through the basement membrane. These results may suggest that the interaction between dermal (animals) and epidermal (animals and humans) melanocytes/melanoblasts and dermal elastin fibers is important for the regulation of melanocyte differentiation. However, it should be confirmed whether human melanoblasts and melanocytes express the elastin-binding protein, and if their differentiation is induced by VGVAPG peptides.

The present results indicate that the immediate increase in melanoblasts and melanocytes after phototherapy and/or mMG is well correlated with the increase in elastin fibers, suggesting the possibility that the elastin fibers are related to the proliferation of melanoblasts and melanocytes. Fibroblast-derived factors [27,28] in addition to keratinocyte-derived factors [29,30] have been reported to regulate the proliferation of melanoblasts and melanocytes. Elastin molecules and VGVAPG peptides are assumed to be one of the fibroblast-derived mitogens toward melanoblasts and melanocytes. The interaction between elastin molecules/VGVAPG and melanoblasts/melanocytes might be important for inducing melanoblast/melanocyte proliferation. Indeed, Chang et al. [25] observed that, during the development of C57BL/6J mouse skin (E9.5, E12.5, E15.5, and E18.5), the increase in the number of epidermal melanoblasts/melanocytes correlated well with the increasing expression of the elastin-binding protein. However, it should be investigated in detail whether, during the development of human skin, the expression of elastin-binding protein correlates with the increase in melanoblasts and melanocytes.

One possible hypothesis as to why the decrease in elastin fibers induces vitiligo and the increase in elastin fibers may induce repigmentation is that the destruction of elastin fibers may promote immune infiltrate (CD8^+^ T cells), resulting in vitiligo development, and the construction of elastin fibers may inhibit immune infiltrate resulting in recovery of vitiliginous skin. 

In the present study, treatment modality differences, namely single vs. double therapy and phototherapy alone vs. phototherapy plus mMG failed to produce any differences in the redifferentiation of melanocytes/melanoblasts and recovery of elastin fibers. After the initiation of the proliferation and differentiation of melanocytes and melanoblasts, phototherapy alone is able to restore melanocyte loss and reduced elastin fibers. However, phototherapy alone may require longer times, and more sessions and doses than phototherapy plus mMG.

Redifferentiation of melanoblasts but not melanocytes in the epidermis of repigmented skin of the young was greater than that of the old, whereas recovery of elastin fibers of the young was less than that of the old. Although this contradictory result cannot be fully explained at present, it might be possible to think that the old can redifferentiate melanoblasts by less recovery of elastin fibers, whereas the young requires greater recovery of elastin fibers to redifferentiate melanoblasts.

## 4. Materials and Methods

### 4.1. Exposures of MEL and NB-UVB

A total of 14 patients (all Japanese, 7 females and 7 males, 6–77 years old) of stable nonsegmental vitiligo vulgaris who possessed white macules in several skin sites visited our clinic from July 2018 to October 2020 (Table 1). Before starting, we explained methods of phototherapy and mMG, and obtained informed consent. We started exposures of 1 to 2 MEDs of MEL (VTRAC, PhotoMedex, Orangeburg, NY, USA) or NB-UVB (Dermary 400, Canon Medical Systems, Tokyo, Japan). The MEL and NB-UVB were exposed to lesional skin alternately because the alternate treatment of either MEL or NB-UVB has been shown to produce an excellent outcome (repigmentation).

### 4.2. Skin Transplantation (mMG)

We usually choose normal scalp skin for the donor skin. The donor skin was produced by exposure to MEL at the dose of 1 to 2 MEDs, 7 days before mMG. Numerous punch biopsies (0.6 to 1.3 mm in diameter, 1.8 mm in depth) of white macules were removed from recipient skin using an electric micro-drill (SIRIUS NT MICRO at 90 g; Gerlach Sirius Nt Micro, Eduard Gerlach GmbH, Luebeck, Germany) as reported previously [16]. Similarly, many punches of normal donor skin (0.6 to 1.3 mm in diameter, 1.5 mm in depth) were taken and placed into holes of lesional skin using fine forceps. After 5 days, we started exposures of 1 to 2 MEDs of either MEL or NB-UVB to all grafts and continued the exposures two or three times a week.

### 4.3. Collection of Skin Samples

Punch biopsies were taken from nonlesional (more than 2 cm apart from the edge of the white macules), lesional, and repigmented skin. Three punch biopsies per each skin site were taken using the same methods as mMG [16]. In the case of phototherapy alone, nonlesional and lesional skin were fixed before phototherapy, while repigmented skin was fixed after full repigmentation. By contrast, in the case of combination therapy of MEL/NB-UVB and mMG, nonlesional and lesional skin were fixed at the time of mMG treatment.

### 4.4. Histochemistry

Methods of histochemistry were reported previously [16]. The number of melanocytes (cells positive to the dopa reaction) and the number of melanoblasts plus melanocytes (cells positive to the combined dopa–premelanin reaction) in the epidermis were counted through 10 consecutive sections (each individual datum was an average of 3 skin samples) and expressed per 0.1 mm^2^ of the interfollicular epidermis. The number of melanoblasts was calculated by subtracting the number of melanocytes from the combined number of melanoblasts and melanocytes. A “melanoblast” is defined here as an unpigmented cell that possesses no tyrosinase activity. Collagen staining (Aniline blue) and elastin staining (Victoria blue) were also reported previously [12].

### 4.5. Statistics

The statistical significance of difference in the number of melanocytes and melanoblasts was determined by Student’s *t*-test (two-tailed). Furthermore, difference in the percentage of recovery of elastin fibers was determined by the nonparametric Mann–Whitney *U* test.

## 5. Conclusions

The present study suggests that the downregulation and upregulation of elastin fibers correlates well with the melanocyte loss and redifferentiation in the epidermis of vitiliginous skin.

## Figures and Tables

**Table 1:** Collagen and elastin fibers in nonlesional, vitiliginous, and repigmented skin after exposures with MEL/NB-UVB and/or mMG in addition to the melanocyte and melanoblast populations/0.1 mm^2^ of interfollicular epidermis.

No.	Gender (Age)	Skin	MEL/NB (No. of Sess)	MEL/NB Month	Dose J/cm^2^	mMG Month	No. of M			No. of Mb			Collagen		Elastin	
							Non	Les	Repig	Non	Les	Repig	Les	Repig	Les	Repig
1.	M (15)	Belly	NB (50)	12	38.3	No	110.9	0	105.8	104.3	0	106.3	→	→	↓↓↓	↑↑↑
2.	M (31)	Chin	NB (70)	10	51.3	No	134.8	0	120.4	117.4	0	82.9	→	→	↓↓	↑↑↑
3.	F (11)	Neck	MEL (8) + NB (8)	4	4.1	4	127.5	0	177.9	103.8	0	103.9	↓	↑	↓↓↓	↑↑↑
4.	M (71)	Neck	MEL (1) + NB (6)	1	10.1	1	53.7	0	82.3	95.0	0	94.0	→	→	↓↓↓	↑↑↑
5.	F (45)	Neck	MEL (5) + NB (6)	0.7	4.5	0.7	87.5	0	91.6	90.0	0	71.1	→	→	↓↓↓	↑↑↑
6.	M (15)	Neck	MEL (3) + NB (1)	2	1.1	2	111.3	0	167.8	98.9	0	150.4	→	→	↓↓	↑↑↑
7.	M (6)	Eyelid	MEL (27) + NB (3)	5	8.0	5	107.8	0	108.3	99.9	0	126.9	→	→	↓	↑↑
8.	M (10)	Head	MEL (35) + NB (31)	10	50.1	10	125.2	0	299.5	100.1	0	283.5	→	→	↓↓	↑↑
9.	F (14)	Eyelid	MEL (4)	1	0.3	No	130.5	0	85.7	102.7	0	96.4	→	→	↓	↑↑
10.	F (13)	Face	MEL (12) + NB (13)	3	7.6	No	93.4	0	126.7	101.2	0	128.6	→	→	↓	↑↑
11.	F (41)	Eyelid	MEL (2) + NB (38)	7	25.5	7	109.9	0	174.1	113.8	0	108.1	→	→	↓↓↓	↑↑↑
12.	F (71)	Arm	NB (51)	24	52.4	No	98.5	0	107.6	103.5	0	97.9	↓	↑	↓↓↓	↑↑↑
13.	F (77)	Hand	NB (151)	12	64.1	No	150.5	0	138.6	107.3	0	99.5	→	→	↓↓↓	↑↑↑
14.	M (65)	Cheek	MEL (12) + NB (12)	2	8.2	2	120.8	0	88.4	106.5	0	89.9	→	→	↓↓↓	↑↑↑
Ave	34.6 ± 6.8		39.2 ± 10.0	6.7 ± 1.7	23.3 ± 5.9		111.6 ± 6.1		133.9 ± 14.9	103.2 ± 1.8		117.1 ± 13.4				

No of session (sess) of MEL (monochromatic excimer light, 308 nm) and/or NB (narrow-band UVB, 311 nm) reached 4 to 151 and were continued for 0.7 to 24 months. Moreover, machine-using minigraft transplantation (mMG) was also performed for 0.7 to 10 months. Non, nonlesional; Les, lesional; Repig, repigmented; Ave, average. There is no statistically significant difference in the average numbers of melanocytes and melanoblasts between Non and Repig. As compared with nonlesional dermis, elastin fibers but not collagen fibers were decreased (14/14 = 100%). However, elastin fibers were completely restored in the dermis of repigmented skin. →, no change; ↓, slightly decreased; ↓↓, decreased; ↓↓↓, decreased dramatically; ↑, light recovery; ↑↑, moderate recovery; ↑↑↑, full recovery and/or stimulation.

**Table 2:** The melanocyte and melanoblast populations in the epidermis of repigmented skin after phototherapy and/or skin transplantation.

	No. of Melanocytes/0.1 mm^2^		No. of Melanoblasts/0.1 mm^2^	
**(A)**	**Single MEL/NB vs Double MEL/NB**			
	Single	Double	Single	Double
	(*n* = 5)	(*n* = 9)	(*n* = 5)	(*n* = 9)
	111.6 ± 7.8	146.3 ± 21.7	96.6 ± 3.4	128.5 ± 19.7
**(B)**	**MEL/NB alone vs** **MEL/NB + mMG**			
	MEL/NB	MEL/NB + mMG	MEL/NB	MEL/NB + mMG
	(*n* = 6)	(*n* = 8)	(*n* = 6)	(*n* = 8)
	114.1 ± 6.9	148.7 ± 24.2	101.9 ± 5.6	128.5 ± 22.2
**(C)**	**Gender**			
	Female	Male	Female	Male
	(*n* = 7)	(*n* = 7)	(*n* = 7)	(*n* = 7)
	128.9 ± 13.0	138.9 ± 26.6	100.8 ± 6.0	133.4 ± 24.6
**(D)**	**Age**			
	Young	Old	Young	Old
	(*n* = 7)	(*n* = 7)	(*n* = 7)	(*n* = 7)
	153.1 ± 25.5	114.7 ± 11.5	142.3 ± 22.7 *	91.9 ± 4.2 *

Monochromatic excimer light (MEL) or narrow band-UVB (NB-UVB); mMG, machine-using minigraft transplantation; Young, from 6 to 15 years; Old, from 31 to 77 years. Each datum is an average ± standard error of the mean. No statistical significant difference was observed except the no. of melanoblasts observed at young and old patients (underlined, * *p* < 0.05).

## Data Availability

Further research data are not shared. All necessary data are available in this paper.
